# Datasets from a research project examining the role of politics in social psychological research

**DOI:** 10.1038/sdata.2018.236

**Published:** 2018-10-30

**Authors:** Domenico Viganola, Orly Eitan, Yoel Inbar, Anna Dreber, Magnus Johannesson, Thomas Pfeiffer, Stefan Thau, Eric Luis Uhlmann

**Affiliations:** 1 Stockholm School of Economics, Stockholm, Sweden; 2 INSEAD, Singapore; 3 University of Toronto, Toronto, Canada; 4 University of Innsbruck, Innsbruck, Austria; 5 Massey University, Auckland, New Zealand

**Keywords:** Psychology, Politics

## Abstract

We present four datasets from a project examining the role of politics in social psychological research. These include thousands of independent raters who coded scientific abstracts for political relevance and for whether conservatives or liberals were treated as targets of explanation and characterized in a negative light. Further included are predictions about the empirical results by scientists participating in a forecasting survey, and coded publication outcomes for unpublished research projects varying in political overtones. Future researchers can leverage this corpus to test further hypotheses regarding political values and scientific research, perceptions of political bias, publication histories, and forecasting accuracy.

## Background & Summary

This document describes in detail the data gathered and analyzed for the paper^1^: “Is research in social psychology politically biased? Systematic empirical tests and a forecasting survey to address the controversy.” The primary aim of the project was to provide empirical tests of potential effects of political values on scientific research. Prior work finds that academics are overwhelmingly liberal, especially when it comes to their values on social issues^2–7^. However, whether—and how—the values of scientists may shape the research itself remains an open question.

In this project we recruited thousands of independent raters, assigned scientific abstracts from social psychology to them, and asked them to assess whether explanations focused more on conservatives rather than liberals (explanatory differences^8^), and whether conservatives were explained in a more negative light than liberals (evaluative differences). Then, we recruited scientists to participate in a forecasting survey testing whether they could accurately predict the directions and the effect sizes of both evaluative and explanatory differences, and further examined whether they updated their beliefs about politics in science in light of the empirical results. Finally, we tracked the number of abstracts that eventually were published and the impact factors of the journals. This allowed to test whether conference abstracts that focus more on conservatives than on liberals, or explain conservative ideology in more negative terms, are more likely to be published, and if so, whether they are also more likely to be published in journals with high impact factors.

The overall project had four components:

1. **Ratings of Political Relevance.** Nine hundred and thirty four independent raters recruited on Amazon Mechanical Turk (MTurk) were asked to evaluate the political relevance of 846 abstracts selected from the ten years of programs of the Society for Personality and Social Psychology annual conference (2003–2013) because they contained politically relevant keywords (e.g., *liberal, conservative*). Abstracts that were evaluated as relevant to politics by at least 60% of the raters comprised the data set for our main study (*N* = 306).
2. **Ratings of Political Content (main study).** Two thousand five hundred and sixty independent raters assessed the content of the 306 politically relevant conference abstracts. Raters reported their judgments of evaluative and explanatory differences regarding conservatism and liberalism in the scientific abstracts as well as demographic characteristics including their own political orientation.
3. **Forecasting Survey.** One hundred and ninety eight scientists, researchers, and others, mainly active in the areas of psychology and sociology, participated in a forecasting survey about the role of politics in the social sciences. Forecasters were asked to make empirical predictions regarding the effect sizes and significance levels of evaluative and explanatory difference as determined in the Rating of Political Content study. In addition to their forecasts, participants were asked to express individual beliefs regarding the amount of political bias in science and on the determinants of any such bias. In order to examine whether forecasters updated their beliefs in light of the evidence, we surveyed participants’ beliefs twice: before and after providing them with the effect sizes for evaluative and explanatory differences from the Ratings of Political Content study.
4. **Likelihood of Publication Study.** We investigated how many of the 306 politically relevant conference abstracts were eventually published. We also collected data about the impact factor of the journals in which the study eventually appeared, as well as the year of publication. We studied whether the political connotations of a conference abstract predict if (and where) the research is eventually published.

In the main data collection, we find significant evaluative and explanatory differences in social psychology research abstracts as assessed by independent raters, with no significant moderation by potentially relevant rater characteristics such as their political orientation. In the scientific abstracts, not only are conservatives explained more than liberals, but they are also explained more negatively. In the forecasting survey, we find that scientists accurately predict the direction of the objective evaluative and explanatory differences, but significantly overestimate both effect sizes. However, forecasters also update their initial beliefs about the role of politics in science after being told the empirical results from the main study. In the likelihood of publication supplementary study, we find no evidence that academic journals are prone to publishing research that focuses more on conservatives or casts them in a negative light.

Table 1 summarizes the main characteristics of each data collection for the Politics in Science project; the rest of the document details the structure and the content of the data used in each of the four studies.

## Methods

### Dataset 1: Ratings of Political Relevance

#### Participants

We collected 846 study abstracts from the SPSP conference programs that were presented between 2003 and 2013 and which contained politics-relevant keywords. Each abstract was further assessed as politically relevant or not by 934 independent raters recruited from Amazon Mechanical Turk (MTurk) - a web-based platform that connects diverse sample of online independent workers with tasks that demand human intelligence^9–11^. Recruitment was restricted to participants from the United States, whom had achieved an approval rate of 95% or higher from employers who had hired them to complete online tasks, and who had completed at least 100 tasks online with a highly satisfactory evaluation from the employer. In addition to their assessments of political relevance, raters reported demographic characteristics including their gender, birth year, income, education, race/ethnicity, political orientation (social, economic, overall, and political party affiliation), and English proficiency (native speaker or, if not a native speaker, at what age English was learned).

#### Methods

Starting with ten years (2003–2013) of online postings from the Social of Personality and Social Psychology (SPSP) annual conference, one of the main academic conferences of the field of social psychology, we collected poster presentations or talks which included the search terms: *liberal, conservative, democrat, republican, politics, political, conservatism,* and *liberalism*. This generated the initial sample of 846 abstracts.

To guarantee a random and isometric assignment of the 846 abstracts we created an algorithm designed to randomly allocate 20 abstracts to each of the 934 independent raters. This resulted in an average of 25.1 allocations and ratings per abstract. Raters were asked to determine the political relevance of each abstract: “Is the research about how political liberals and conservatives think, about differences between political liberals and conservatives, about differences in opinion on a political issue, about which liberals and conservatives typically have different opinions, or about voting or other political behavior? (Yes/No).” Abstracts classified as politically relevant by more than 60% of raters were retained for the main study, yielding 306 conference abstracts.

The complete survey materials are provided in Supplement 1 of the published report of the project^1^.

The full dataset for the Ratings of Political Relevance study is publicly available in the file “Participants_Excel_study 1.xlsx” (Data Citation 1).

### Dataset 2: Ratings of Political Content

#### Participants

A separate group of 2,560 MTurk raters were recruited to assess the 306 politically relevant abstracts for evaluative and explanatory differences regarding conservatives and liberals. We used similar criteria to recruit raters as before: participants from the United States, with a record approval rate of 95% or higher, and whom had previously completed at least 100 tasks online with a highly satisfactory score from their employer. In addition to providing their assessments of the political content of the abstracts, raters reported demographic characteristics including their gender, age, income, education, race/ethnicity, political orientation (social, economic, overall, and political party affiliation), and English proficiency. Finally, we asked participants to report whether they read the abstracts carefully (emphasizing that there was no penalty for answering “no”).

#### Methods

Starting with the 306 politically relevant abstracts identified in Study 1, we generated 2,560 different survey variations each containing a subset of the abstracts. We used an algorithm to ensure random sequencing and allocation of abstracts to each independent rater. The final dataset captured 48,960 recorded responses from raters.

The abstracts text presented in the study excluded abstract titles, publication year and any personal information regarding the authors. Two thousand five hundred and sixty online independent raters each assessed 20 abstracts, the exception being a subset of 160 raters who rated only 6 abstracts since the study examined a total of 306 abstracts. The order in which ratings of the abstracts for their characterizations of conservatives and liberals were done (conservatism ratings first or liberal ratings first) was counterbalanced between-subjects. Abstracts were assessed for either evaluative or explanatory differences, leading to a 2 (explanatory differences ratings vs. evaluative differences ratings) by 2 (order of items: political conservatism ratings first or political liberalism ratings first) between-subjects design.

To measure explanatory differences the participants were presented with the following questions: “To what extent does this research attempt to explain political liberalism or liberal ideas?” and “To what extent does this research attempt to explain political conservatism or conservative ideas?” (1 = not at all, 7 = a great deal). To measure evaluative differences, the participants were presented with the following questions: “How does this research characterize political liberals or liberal ideas?” and “How does this research characterize political conservatives or conservative ideas?” (1 = extremely negatively, 4 = neutral, 7 = extremely positively). Separate groups of raters assessed evaluative and explanatory differences for each abstract. Hence, for each abstract, we could compute four aggregated scores: evaluative tendencies towards conservatives, evaluative tendencies towards liberals, explanatory tendencies towards conservatives, and explanatory tendencies towards liberals. We found significant evaluative and explanatory differences in the abstracts, such that conservatives were more often the target of explanation and were explained in a more negative light than liberals.

The political orientation of the raters (liberal or conservative) did not drive their assessments of the abstracts. Contrary to expectations, politically conservative raters were, if anything, less likely than liberal raters to perceive scientific abstracts as focusing on conservatives as the target of explanation and as characterizing conservatism negatively. Importantly, however, both conservative raters (5–7 on a 1–7 scale of political orientation) and liberal raters (1–3 on the same scale) perceived the abstracts as biased, and in the same direction.

The complete survey materials are provided in Supplement 2 of the published report of the project^1^.

The full dataset for the Ratings of Political Content study is publicly available in the file “bias rating results-Analysis.csv” (Data Citation 1).

### Dataset 3: Forecasting Survey

#### Participants

The forecasting survey was advertised mainly to academic audiences with help from academics with large Twitter followings, by using Facebook pages such as Psych Map and the Psychological Methods Discussion group, and by posting invitations on the SPSP listserv and Social Psychology Network. The social media advertisement materials are provided in Supplement 5 of the published report of the project^1^. Forecasters were not paid to participate in the survey. There were no restrictions on participation based on nationality, education or occupation, although we were mainly interested in the predictions of academics.

The vast majority of the participants completed the survey in less than 15 min, however, in few cases, the completion time exceeded one hour. Three hundred and nine participants started the survey, 107 participants did not finish it, and 4 opted for withdrawal post-completion. Hence, the final sample size for the forecasting survey is 198. 93.4% of the participants that completed the survey (185 out of the 198) were working in academia at the time of the study. Table 2, 3, 4 summarize the main fields of research of forecasters, their academic job rank, and their country of origin, respectively. Note that only aggregate statistics are provided for these three variables (field of study, job rank, and nationality) to preserve participant anonymity.

This is complemented by individual-level data on forecasters including their age (*M* = 36, *SD* = 10.9), gender (120 men, 79 women), and highest level of education completed (128 have a doctoral degree, 3 have a professional degree, 50 have a masters degree, 14 have a bachelor degree in college, 1 has some college without completing the degree, 1 has a high school degree). As detailed in the published report of the project^1^, demographic characteristics of forecasters were generally not associated with predictive accuracy.

#### Methods

Participants accessed the survey by clicking the link they found on Facebook, Twitter, or on psychology dedicated websites. First, participants were given more information about the goal of the project, namely, to predict the results in terms of Cohen’s d of a study (the Ratings of Political Content study) assessing two potential political effects in scientific research: evaluative differences and explanatory differences. Then, they were provided with all the details of the study and with the definition of the Cohen’s d statistic. Participants then made their forecasts for the effect size and the direction of the effect for both explanatory differences (conservatives explained directionally more often vs. liberals explained directionally more often), and evaluative differences (conservatives explained directionally more negatively vs. liberals explained directionally more negatively).

**Figure 1:**
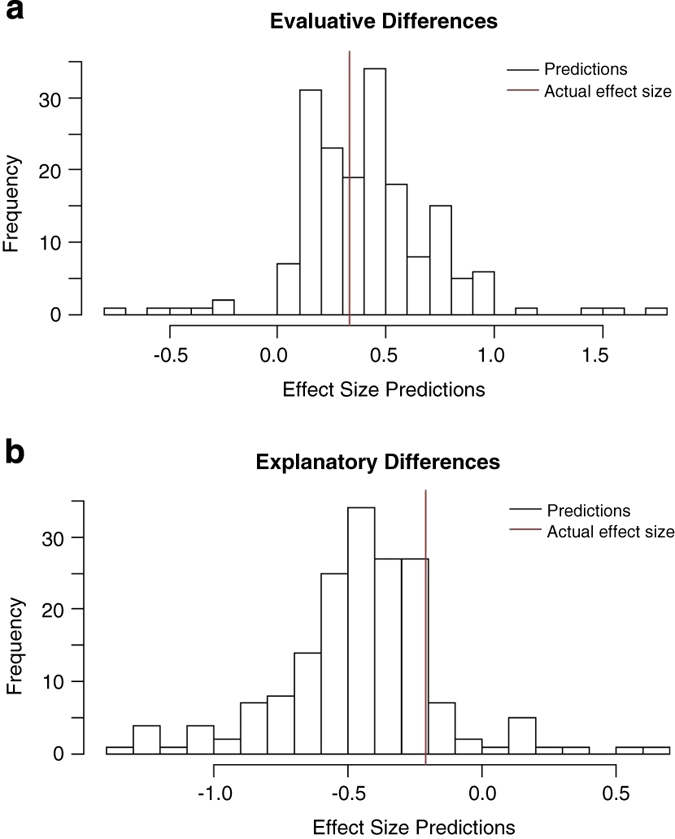
Participants’ forecasts and the actual effect sizes. (**a**) Distribution of evaluative differences. (**b**) Distribution of explanatory differences.

The forecasters predicted explanatory differences such that conservatives would be explained more (effect size −0.41), and an evaluative differences such that conservatives would be characterized more negatively (effect size 0.45); the directions match the obtained effect sizes in the Ratings of Political Content study (−0.21 for explanatory differences and 0.33 for evaluative differences), although both effect sizes were significantly overestimated by forecasters. Histograms in Fig. 1a and b (see also^1^) provide graphical summaries of forecasters’ predictions and the actual effect sizes for evaluative and explanatory difference.

**Figure 2:**
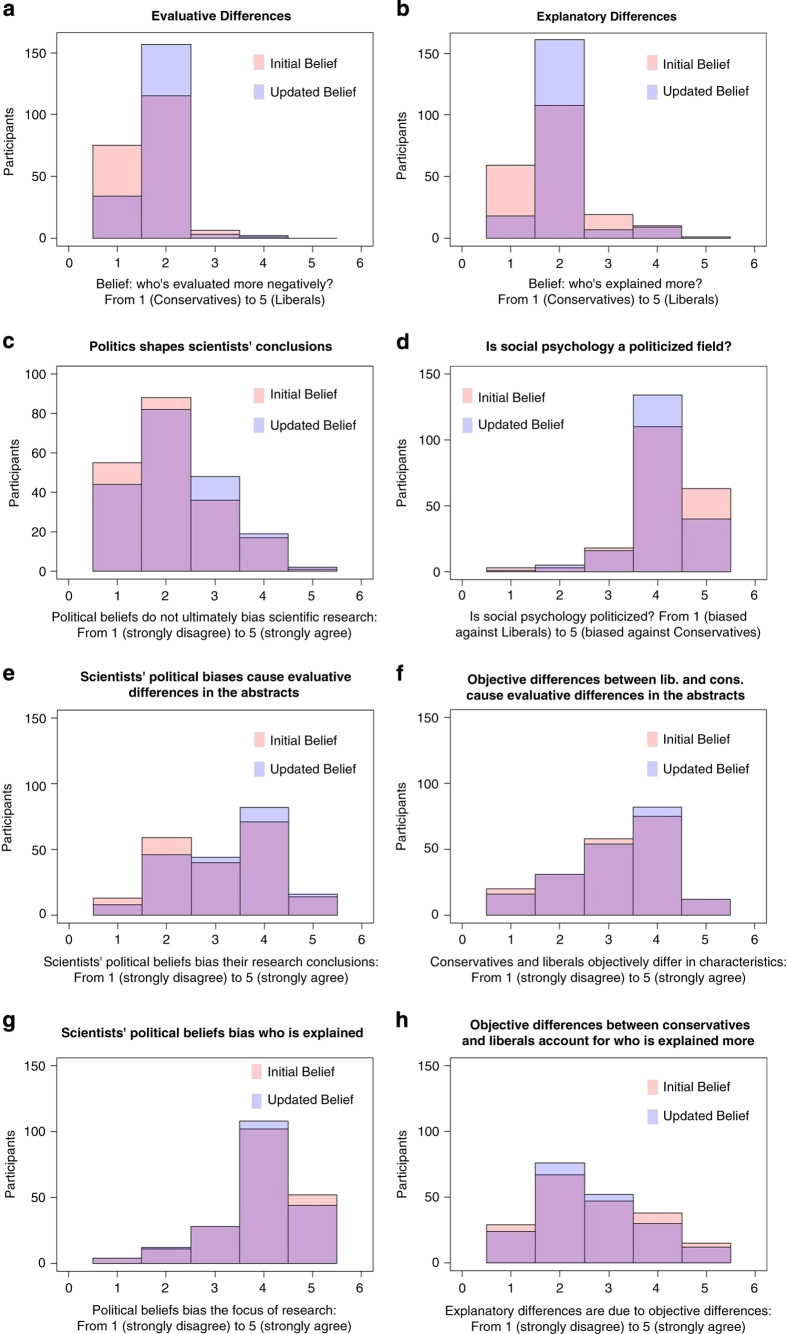
Participants’ original beliefs and updated beliefs after learning the empirical results. (**a**) Beliefs about evaluative differences. (**b**) Beliefs about explanatory differences. (**c**) Beliefs about politics shaping scientists’ conclusions. (**d**) Beliefs about social psychology being a politicized field. (**e**) Beliefs about scientists’ political biases causing evaluative differences in the abstracts. (**f**) Beliefs about objective differences between liberals and conservatives causing evaluative differences in the abstracts. (**g**) Beliefs about scientists’ political views generating bias about who is explained. (**h**) Beliefs about objective differences between conservatives and liberals accounting for who is explained more.

In addition, forecasters were asked a set of questions aimed at eliciting their beliefs about the role of scientists’ political values in scientific research (i.e., social psychology evaluates conservatives and liberals differently; social psychology seeks to explain conservatism and liberalism to different degrees; personal beliefs of social scientists do not influence the conclusions of their research; social psychology is a political neutral field or generally biased; evaluative/explanatory differences are due to scientists’ political beliefs and/or objective differences between conservatives and liberals) on scales ranging between 1 and 5. The same set of questions was asked twice, both before and after the actual effect sizes obtained in the Ratings of Political Content study were disclosed. Belief updating is inferred by comparing initial beliefs to beliefs after effect sizes are disclosed; Fig. 2a through 2h provide a full set of graphs summarizing belief change over time. Finally, participants reported demographic information including their political orientation.

To investigate whether social scientists believe that research in social psychology exhibits evaluative and explanatory differences with regard to conservatives and liberals, we tested whether the average of the distribution of individual forecasts is significantly different from zero. We were also interested in understanding whether scientists can correctly predict the effect sizes for evaluative and explanatory differences. We therefore computed a z-test on the average predicted directional effect sizes from the forecasting survey being different from the obtained effect size from the main study. We ran multiple variable linear regressions to test whether individual characteristics of the forecasters (political ideology, gender, age, education, and working in academia) were correlated with higher accuracy of predictions, measured by absolute prediction error, and with the predicted evaluative and explanatory differences between conservatives and liberals.

To test whether survey participants update their beliefs about politics in science after learning the objective effect sizes for evaluative and explanatory differences from the Ratings of Political content study, we ran a set of paired t-tests. From this we can conclude that scientists updated their beliefs in light of the empirical evidence, moderating their views about the degree of political bias in science.

The complete survey materials are provided in Supplement 3 of the published report of the project^1^.

The full dataset is publicly available in the file “Forecasting_Survey_Anonymous.csv” (Data Citation 2).

### Dataset 4: Likelihood of Publication study

#### Participants

The final phase of the project was aimed at assessing whether institutional gatekeepers such as reviewers and editors screen out politicized research or favor its publication. We contacted via email the authors of the 306 conference abstracts selected for the Ratings of Political Content study, rather than looking for the abstract and title in scientific databases as these are often subject to change between conference presentation and publication. We started by contacting first authors, then last authors, then from second author down in descending order. We asked authors to indicate whether the work was published and if so, in which journal. Out of the total of 306 conference abstracts, we received information about 137 of them, indicating that 47% of the articles were published (64 out of 137). We collected the 1-year impact factor of each journal which published at least one article out of the 64 for which we received the authors’ feedback. We collected information about the 1-year impact factor checking the front page of the journals’ websites. When information about 1-year impact factor was not available, we collected the 5-year impact factor instead: this happened in 3 cases. However, for 4 journals we did not manage to find any information about their impact factor, and one article was published in a book. Hence, we managed to find information about impact factors for 56 journals out of 64.

#### Methods

For the Likelihood of Publication study, we used two datasets: the one collected for the Ratings of Political Content study and the one containing information on publication status and impact factors described in the previous section. For each abstract we computed two difference scores from the Ratings of Political Content study, one for evaluative differences and one for explanatory differences. We merged these difference scores with the dataset containing information on publication status of the conference abstracts and the impact factors of the journals in which abstracts eventually ended up being published. This allowed us to match the scores regarding evaluative and explanatory differences in each abstract with its publication status.

This combined dataset allowed us to investigate whether the politics of a conference presentation predicts whether or not the research eventually gets published in an academic journal, and in how prestigious an outlet. We found no relationship between explanatory and evaluative differences in the abstracts and either their publication status or the impact factor of the journal.

The complete materials from the survey of original authors are provided in Supplement 6 of the published report of the project^1^.

The full dataset is publicly available on OSF platform (Data Citation 3) in the file “gatekeeper_all_abstracts.csv”. In addition to publication status of the conference abstract and impact factor of the journal, the dataset also contains information about the year in which the research was presented at the SPSP annual conference.

## Data Records

The four datasets are available on the Open Science Framework (OSF) platform. All records were anonymized in conformance with privacy protection standards and with the informed consent forms accepted by each participant. In particular, for the dataset used in the Ratings of Political Relevance and Rating of Political Content study, we anonymized MTurk participants’ unique identification number. For the Forecasting survey dataset, we anonymized email addresses, IP addresses, start and end date of the survey, ethnicity, country of birth, country of residence, years of experience with English, job rank and department of affiliation, geographical information about the place in which the survey was taken, and in general all answers that involved free text to be inserted by the participant. For the Likelihood of Publication study, we anonymized author names and the email addresses used to contact the authors, the day in which we sent the emails, the journals’ names and website addresses, and any reference to the title/content of the abstracts. We did not include the full text of the abstracts for confidentiality reasons, since full text abstracts could easily be used to recover authors’ identities.

The four datasets are accompanied by a “variables codebook” excel file which gives a detailed description of all the variables. The codebook provides information on variable names, the survey questions from which the variables are obtained when relevant, the variable coding and some additional details, as well as a brief methodological overview of the four studies. The analyses were conducted using the R language^12,13^ and environment for statistical computing (2014) available at the following URL: https://www.r-project.org/. R files with the codes to replicate the analysis (one for each part of the project) are provided in the R code files folder in the OSF platform. In total, we make publicly available four datasets, R codes for the analyses, and a codebook file for all the variables we used.

Dataset 1 – The data file “Participants_Excel_study 1.xlsx” can be accessed on the OSF platform (Data Citation 1). This includes 846 abstracts rated by 934 independent raters recruited using the Mechanical Turk platform. The file also contains the rater’s demographic characteristics: gender, birth year, income, education, race/ethnicity, political orientation (social, economic, overall, and political party affiliation), and English proficiency, as well as the independent ratings used to select the 306 politically relevant abstracts for the main study.

Dataset 2 – The data file “bias rating results-Analysis.csv” is located on the OSF platform (Data Citation 1). It consists of 195,840 ratings by 2,560 independent raters recruited using the Mechanical Turk platform. This file contains the raters’ demographic characteristics such as their political views on economic and social issues, as well as their political party preferences. Most importantly, the dataset includes their ratings regarding explanatory/evaluative differences in the scientific abstracts.

Dataset 3 – The data file “Forecasting_Survey_Anonymous.csv” is located on the OSF platform (Data Citation 2). It contains the records of the 304 participants in the forecasting survey. The dataset is composed of 63 variables. These include the predicted size and direction (in favor of conservatives or in favor of liberals) for explanatory differences and evaluative differences; a set of 22 variables concerning beliefs about the role of politics in scientific research; demographics of the participants, such as their age, ethnicity, gender, political preferences, years of experience with English language, role in academia (if any), and education; and finally a set of technical variables for the survey (e.g. the timing of the questions).

Dataset 4 – The data file “gatekeeper_all_abstracts.csv” is publicly available on the OSF platform (Data Citation 3). It contains the available information about the publication status of the conference abstracts. We managed to gather this information for 137 abstracts out of the total of 306. Each abstract is coded as either published or not. If published, we recorded the impact factor of the journal.

## Technical Validation

The datasets “Participants_Excel_study 1.xlsx”, “bias rating results-Analysis.csv”, “Forecasting_Survey_Anonymous.csv” and “gatekeeper_all_abstracts.csv” are the anonymized versions of the datasets from the politics in science project. The preparation of the raw datasets for the final analyses encompasses aggregation and averaging of different survey items from the same scale, reverse coding of certain items, and selection of participants based on proficiency with English, attention paid during the survey, and completion of the survey. The preparation process for each dataset is detailed in the pre-registered analysis plans and in the accompanying R codes with the full analyses.

## Additional information

**How to cite this article**: Viganola, D. *et al.* Datasets from a research project examining the role of politics in social psychological research. *Sci. Data*. 5:180236 doi: 10.1038/sdata.2018.236 (2018).

**Publisher’s note**: Springer Nature remains neutral with regard to jurisdictional claims in published maps and institutional affiliations.

## Supplementary Material



## Figures and Tables

**Table 1:** Study Characteristics.

	**Ratings of Political Relevance**	**Ratings of Political Content (main study)**	**Forecasting Survey**	**Likelihood of Publication Study**
**Type of Study**	Cross-sectional study	Cross-sectional study	Forecasting and belief updating	Correlational study
**Measurement Made**	Judgements	Judgements	Predictions and beliefs	Publication status and impact factors
**Data Collection Method**	Survey method	Survey method	Survey method	Survey method, collection of data from websites
**Study Participants/Observations**	MTurk independent raters	MTurk independent raters	Scientists	Academic abstracts and journals

**Table 2:** Forecasters’ fields of research.

**Department and Field of Research**	**Number of Participants**
Psychology	73
Sociology	59
Marketing	9
Management	9
Economics	8
Political and Public Policy	7
Education	3
Medicine	2
Other Departments	5
Note: 10 participants did not specify their department; ‘Other Departments’ include Anthropology, Communication, Decision Sciences, Linguistics, Media Studies; 13 participants were not academics.	

**Table 3:** Forecasters by academic job rank.

**Job Rank**	**Number of Participants**
Full Professor	21
Associate Professor	28
Assistant Professor	31
Post-doctoral Researcher	28
Lecturer (non-tenure track)	10
Graduate Student	52
Master Student	3
Lab Manager and Research Assistant	5
Note: 7 participants did not specify their job rank in academia; 13 were not academics.	

**Table 4:** Forecasters by country of origin.

**Nationality**	**Number of Participants**
United States	119
Canada	13
Germany	11
The Netherlands	5
United Kingdom	5
Sweden	4
Other Countries	32
Note: 9 participants did not specify their country of origin; ‘Other Countries’ include Argentina, Australia, Austria, Belgium, Brazil, Chile, China, Croatia, Cyprus, France, Hungary, India, Iran, Israel, Italy, New Zealand, Norway, Poland, Portugal, Russia, South Africa, South Korea, and Turkey.	
